# High-throughput 5′P sequencing enables the study of degradation-associated ribosome stalls

**DOI:** 10.1016/j.crmeth.2021.100001

**Published:** 2021-04-02

**Authors:** Yujie Zhang, Vicent Pelechano

**Affiliations:** 1 SciLifeLab, Department of Microbiology, Tumor and Cell Biology, Karolinska Institutet, Solna 171 65, Sweden

**Keywords:** RNA degradation, ribosome stalls, degradome, co-translational decay

## Abstract

RNA degradation is critical for gene expression and mRNA quality control. mRNA degradation is connected to the translation process up to the degree that 5′-3′ mRNA degradation follows the last translating ribosome. Here, we present an improved high-throughput 5′P degradome RNA-sequencing method (HT-5Pseq). HT-5Pseq is easy, scalable, and uses affordable duplex-specific nuclease-based rRNA depletion. We investigate *in vivo* ribosome stalls focusing on translation termination. By comparing ribosome stalls identified by ribosome profiling, disome-seq and HT-5Pseq, we find that degradation-associated ribosome stalls are often enriched in Arg preceding the stop codon. On the contrary, mRNAs depleted for those stalls use more frequently a TAA stop codon preceded by hydrophobic amino acids. Finally, we show that termination stalls found by HT-5Pseq, and not by other approaches, are associated with decreased mRNA stability. Our work suggests that ribosome stalls associated with mRNA decay can be easily captured by investigating the 5′P degradome.

## Highlights

• HT-5Pseq facilitates high-throughput investigation of the 5′P degradome
• Gene-specific ribosome stalls at termination are common and actively regulated
• Termination stalls identified by HT-5Pseq are associated with decreased mRNA stability

## Motivation

Investigating 5′phosphorylated mRNA degradation intermediates is key to understanding 5′-3′ co-translational mRNA decay and ribosome dynamics; however, current approaches are time-consuming and costly. Here, we present an improved high-throughput 5′P degradome RNA sequencing method (HT-5Pseq) that provides in vivo information of ribosome footprints associated with decay at low costs and significantly reduced hands-on time.

## Graphical abstract

**Figure:**
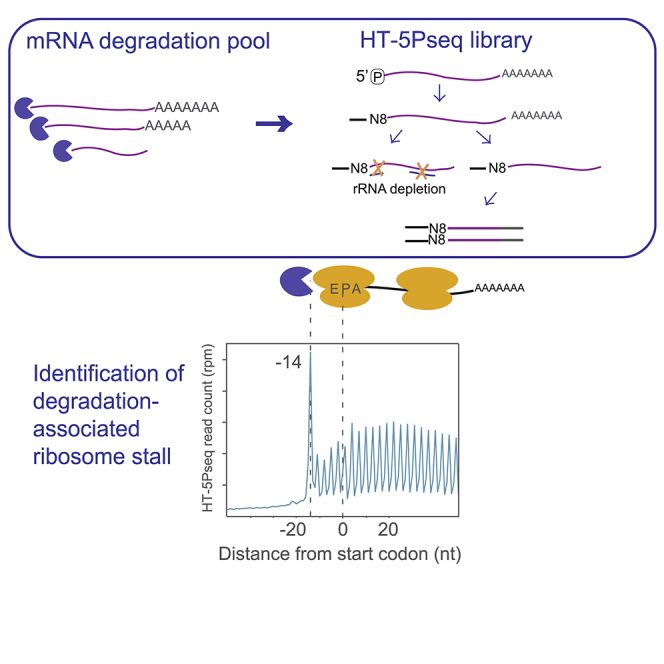

## Teaser

Zhang et al. develop an improved high-throughput 5′P degradome RNA-sequencing method (HT-5Pseq). This method facilitates identification of *in vivo* ribosome stalls associated with mRNA decay during translation.

## Introduction

RNA degradation is an integral player in gene expression, as its balance with RNA synthesis is what determines final mRNA abundance. Thus, to understand gene expression, it is important to explore not only mRNA synthesis but also the factors that shape mRNA degradation. Investigating the RNA degradome is important to understand, for example, differential sensitivity to specialized RNA decay pathways (Nagarajan et al., 2019) or endonucleolytic cleavage (German et al., 2008). In addition to these specialized degradation mechanisms, general processes such as translation are also key to shaping the mRNA degradome (Hu et al., 2009; Pelechano et al., 2015). By investigating 5′ phosphorylated (5′P) mRNA degradation intermediates, we have previously demonstrated that *in vivo* 5′-3′ co-translational degradation provides information regarding ribosome dynamics and codon-specific ribosome stalls. Specifically, in budding yeast, the 5′-3′ exonuclease Xrn1p follows the last translating ribosome, generating an *in vivo* footprint of its position (Pelechano et al., 2015). A similar phenomenon has also been described in other organisms such as *Arabidopsis thaliana* and rice (*Oryza sativa*) (Hou et al., 2016; Yu et al., 2016). We have previously shown that the *in vivo* ribosome footprint as measured by 5′P degradome sequencing (5Pseq) is a good proxy for general ribosome positions (Pelechano et al., 2015; Pelechano and Alepuz, 2017). However, as 5Pseq focuses on those ribosome footprints associated with mRNA degradation, in principle, it should be enriched in those ribosome stalls leading to mRNA decay. Genome-wide ribosome stalls are identified by the accumulation of ribosome-protected mRNA fragments (footprints) at a particular position. However, an increased density of ribosome footprints can be associated with both transient pauses and stable ribosome stalls (Karousis et al., 2020), and those stalls might be stable in the cell or associated with mRNA decay. General methods such as ribosome profiling investigate the composite of all ribosomes in the cell independent of the functional consequences of those stalls, and even optimized ribosome profiling approaches focusing on disomes identify ribosome stalls that are not necessarily associated with decay (Meydan and Guydosh, 2020). However, It is well known that factors such as low codon optimality and ribosome stall often trigger RNA decay (Presnyak et al., 2015; Simms et al., 2017). All this shows the need for an optimized method that is able to identify ribosome stalls prone to decay. Nevertheless, despite our ability to investigate the 5′P mRNA degradome, current sequencing approaches are still cumbersome. Methods originally developed to investigate miRNA cleavage such as parallel analysis of RNA ends (PARE) (German et al., 2009; Hou et al., 2016; Schon et al., 2018) and genome-wide mapping of uncapped and cleaved transcripts (GMUCT) (Yu et al., 2016) are labor intensive and require multiple ligation steps, purification, and gel-size selection. Even our original 5Pseq protocol requires multiple enzymatic steps and bead-based purifications, which limits its application to investigate hundreds of samples (Pelechano et al., 2016).

In addition to investigating the presence of 5′P mRNA degradation intermediates, it is important to capture their full diversity. Cytoplasmic mRNA decay in eukaryotes is often initiated through deadenylation (Brewer and Ross, 1988; Garneau et al., 2007). After poly(A) tail shortening by deadenylase complexes PAN2/3 and CCR4-NOT, mRNA can be degraded exonucleolytically in either 5′-3′ or 3′-5′ orientation. The existing diversity regarding 5′P mRNA degradation intermediate poly(A) length suggests that multiple subpopulations of degradation intermediates co-exist. We have previously shown that both poly(A) tail enrichment and rRNA depletion can be used to identify mRNA degradation intermediates (Pelechano et al., 2015, 2016). However, identifying subpopulations with and without a poly(A) tail in the same experiment and enabling their parallel analysis will offer a more general view of the mRNA degradome.

To facilitate the investigation of the 5′P mRNA degradome in relation to the translation process, we have developed a high-throughput 5′P-sequencing approach called HT-5Pseq. This approach is flexible, cheap, and scalable. We benchmark HT-5Pseq against 5Pseq and investigate its applicability in *S. cerevisiae* and *S. pombe*. We demonstrate that it offers high-quality degradome information at a fraction of the cost of 5PSeq and with significantly decreased hands-on time. Additionally, by differentially capturing those partially deadenylated intermediates of degradation, it improves the resolution around the stop codon. We use this approach to compare ribosome stalls at termination level among different growth conditions and show that they are actively regulated. Finally, we investigate the functional consequences of those stalls by comparing HT-5Pseq with ribosome profiling and disome sequencing (disome-seq). We focus on those features frequently associated with degradation-associated ribosome stalls and investigate their relationship with mRNA stability.

## Results

### Development of HT-5Pseq for investigation of 5′-phosphorylated mRNA degradation intermediates

**Figure 1:**
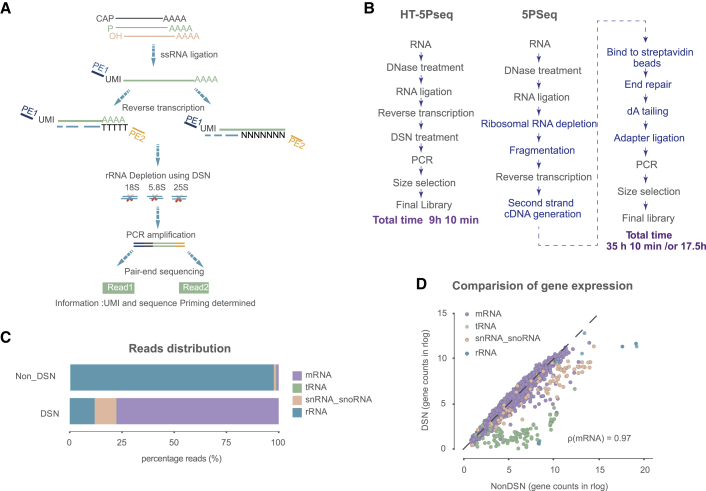
Development of HT-5Pseq (A) Outline of HT-5Pseq method . A specific RNA oligo (PE1) is ligated to 5′P RNA molecules. RNA is reverse transcribed by using a mix of sequencing oligos (PE2) containing oligo(dT) and random hexamers. The cDNA originating from rRNA is depleted using DNA oligos and double-strand specific nuclease (DSN). cDNA is PCR amplified and sequenced. (B) Flowchart of HT-5Pseq and 5Pseq for each step. Different and eliminated steps in 5Pseq are marked in blue. (C) Improvement of mRNA mappability in *S*. *cerevisiae* HT-5Pseq after rRNA depletion. NonDSN refers to control libraries omitting rRNA depletion probes. (D) Differential gene-specific 5′P read coverage.

To enable the high-throughput investigation of co-translation mRNA degradation and ribosome dynamics, we set out to improve our previous 5Pseq approach (Pelechano et al., 2016). We identified multiple bottlenecks for its systematic application: the high number of enzymatic reactions, the required DNA purification steps, and the difficulty associated with rRNA depletion (that contain the majority of the 5′P RNAs in the cell). By improving these steps, we decreased both the required hands-on time and the cost per library. In HT-5Pseq, we first ligate an RNA oligo containing unique molecular identifiers (UMIs) to the 5′P ends of total RNA (Figures 1A, 1B, and S1A). Total ligated RNA is subjected to reverse transcription using a mix of Illumina-compatible oligos priming with a random hexamer and oligo(dT). This enables the capture of 5′P sites independent of their distance to the mRNA 3′ end and facilitates the recovery of regions proximal to the poly(A) site that are not efficiently reverse transcribed by using random hexamers alone. The direct priming with Illumina-compatible oligos also obviates the necessity of *in vitro* RNA fragmentation and associated clean-up of beads that was required in our previous 5Pseq implementation (Figures 1B and S1A). Also, by combining random hexamer and oligo(dT) priming (Xu et al., 2009) we ensured a more homogeneous read coverage along the mRNA and facilitate the priming of regions proximal to the poly(A) tail, which in turn improves sequencing coverage around the stop codon region (see below). After the generation of cDNAs containing Illumina-compatible adaptors at both sides, we degraded RNA with NaOH to generate a single-stranded cDNA library. To selectively deplete abundant cDNA complementary to rRNA, we designed a pool of affordable non-modified DNA oligos (Table S1A). We annealed the rRNA depletion oligos with the single-stranded cDNA library at 68°C and treated the mix with duplex-specific nuclease (DSN) (Zhulidov, 2004), a strategy that has been previously shown to be useful in depleting problematic sequences from RNA sequencing (RNA-seq) at the cDNA stage (Archer et al., 2014). After this step, the rRNA-depleted cDNA library is purified and PCR amplified to generate the final sequencing library. By eliminating the need for costly biotin-based rRNA depletion and time-consuming mRNA fragmentation (Figures 1B and S1A), we reduced the number of bead-based purifications (from 10 to 4). Also, by adding a second Illumina adaptor (PE2) during the reverse transcription, we eliminated the enzymatic steps required for end-repair, dA-tailing, and DNA ligation. This improvement also obviates the need for additional beads-based clean-up steps that often lead to sample loss. In summary, all these modifications reduced the total time from 35 h to 9 h, and reduced the cost of library preparations by 70% (Figure S1A and Table S2).

The main application of HT-5Pseq is the identification of translation pausing associated with degradation. However, to achieve sufficient sequencing coverage to perform such analysis, it is important to deplete first those 5′P ends associated with the rRNA. DSN-based rRNA depletion increases the percentage of reads aligned to mRNAs from 1.1% to 77.6% (Figure 1C) as well as the associated sequencing library complexity (Figure S1B). Accordingly, DSN treatment also decreases the rRNA reads percentage from 98.0% to 12.0% (Figure 1C). We also confirmed that DSN treatment does not compromise HT-5Pseq reproducibility (Figures S1C–S1E) and has a limited effect on mRNA coverage (Figure 1D). DSN treatment decreases sequencing coverage of the depleted rRNA regions (in blue) and other features with significant secondary structure such as tRNAs (in green). Only a few mRNAs (0.57%, 31 of the 5,398 detected) present significantly decreased coverage, likely caused by their ability to form hairpins at 68°C. The main advantage of using a DSN-based rRNA depletion approach (Chung et al., 2015) is the simple and flexible design of rRNA depletion probes and greater affordability in comparison with commercial rRNA depletion using biotinylated oligonucleotides. To show the flexibility of our rRNA depletion approach, we used probes originally designed for *S*. *cerevisiae* (Table S1A) to investigate the 5′P degradome in the distant yeast *S*. *pombe*. We confirmed that this treatment increased the percentage of usable mRNA molecules, from 5.0% unique mRNA degradation intermediates to 62.3% after DSN treatment (Figures S1F–S1G), even using a suboptimal rRNA depletion panel. All these confirm that HT-5Pseq is a flexible protocol that can be easily adapted to the organism of interest.

### HT-5Pseq provides single-nucleotide resolution insights into 5′-3′ co-translational decay

**Figure 2:**
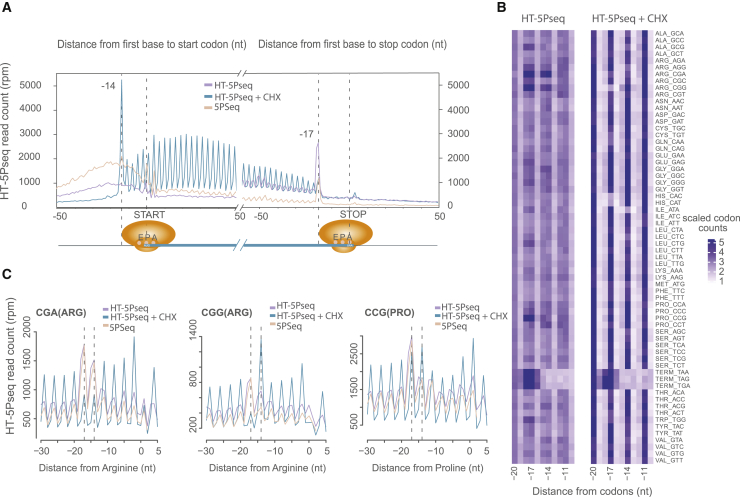
HT-5Pseq reveals ribosome dynamics at codon resolution (A) Metagene analysis for 5′P read coverage relative to open reading frame (ORF) start and stop codon. Cells grown in rich medium (YPD) by HT-5Pseq are shown in purple, cells treated with cycloheximide (CHX) for 5 min in blue, and 5Pseq cells in pink. (B) Heatmap for codon-specific 5′P coverage. Positions −17, −14, and −11 nt represent ribosome protection at the A, P, and E site, respectively, with or without CHX treatment. For each codon, reads were normalized using the total 5′P reads between −30 and 5 nt. (C) 5′P reads coverage for rare arginine (CGA, CGG) and proline codons (CCG). Dotted lines at −17 and −14 correspond to the expected 5′ end of protected ribosome located at the A site or P site, respectively.

Once we confirmed its general applicability, we verified HT-5Pseq’s ability to identify ribosome stalls at single-nucleotide resolution (Pelechano et al., 2015, 2016). As expected, HT-5Pseq recovers the characteristic 3-nt periodicity associated with 5′-3′ co-translational mRNA degradation (Figure 2A). To further confirm its sensitivity, we investigated codon-specific ribosome stalling by using our newly developed computational pipeline *Fivepseq* (Nersisyan et al., 2020). We clearly identified pauses associated with slow codons such as arginine (encoded by CGA, CGG) and proline (encoded by CCG) (Figures 2B and 2C) as we and others have previously shown (Pelechano and Alepuz, 2017; Schuller et al., 2017). Reassuringly, we observed codon-specific pauses in HT-5Peq similar to those observed when the same sample was analyzed by using our previously developed 5Pseq (Figure 2C).

To demonstrate the ability of HT-5Pseq to detect direct perturbations of the translation process, we investigated mRNA degradome changes after cycloheximide (CHX) treatment. As expected, CHX clearly increases the observed 3-nt periodicity, especially at the 5′ regions of the genes (Figure 2A). In addition to its general effect inhibiting translation elongation, CHX can also mask *in vivo* ribosome pausing at the codon level (Hussmann et al., 2015). As in ribosome profiling, in HT-5Pseq rare codons present higher 5′P protection indicating ribosome stalling, which was lost after CHX treatment (Figure S2A). Accordingly, we observe that the protection pattern for proline (CCG) and arginine (CGG) is displaced one codon after CHX treatment (Figure 2C). We also observed increased protection for codons specifically pausing at A, P, or E sites (Figure 2B). To investigate *in vivo* ribosome dynamics in a different species, we treated *S*. *pombe* with CHX and observed a clear increase in the 3-nt periodicity (Figure S2C), as expected by the general translation inhibition (Figure S2B). As in *S*. *cerevisiae*, rare codons such as CCG (proline) and CGG (arginine) showed higher enrichment without CHX treatment, but lost enrichment at site A in CHX samples (Figure 2C). We also found displaced CGG (arginine) codon from site A to site P in *S*. *pombe* (Figure S2D). Our results thus confirm that HT-5Pseq offers codon-specific information about the co-translational mRNA decay process.

### Regulation of ribosome termination pause associated with degradation

**Figure 3:**
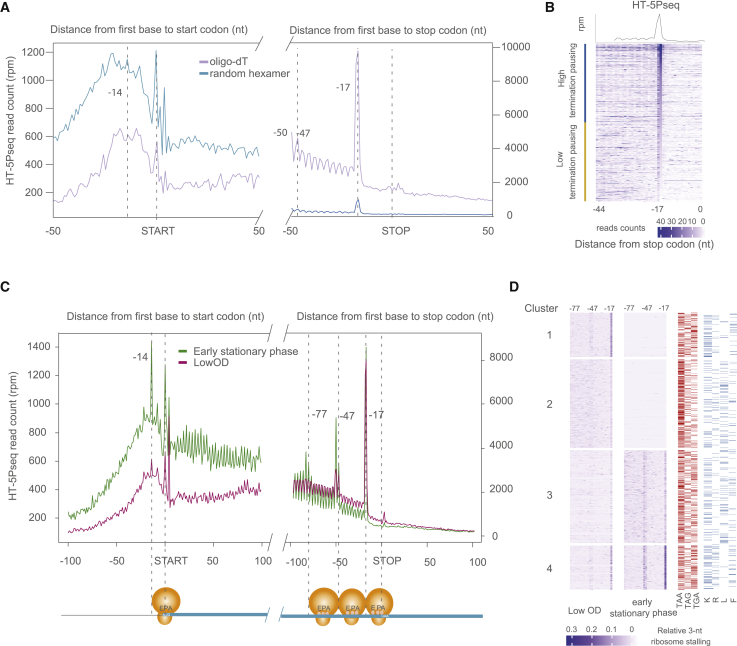
Regulation of termination associated ribosome pauses (A) Metagene analysis for 5′P read coverage relative to ORF start and stop codon for HT-5Pseq reads primed by oligo(dT) (purple) or random hexamer (blue). (B) Gene-specific 5′P read coverage relative to ORF stop codon. Genes are sorted by the relative ribosome protection at the stop codon (−17) with respect to the upstream region (−44 to −17 nt). Only genes with at least 10 rpm (reads per million) in this region were considered. The top 50% genes were defined as the high termination pausing genes and the bottom half were defined as low termination pausing genes. (C) Metagene analysis for 5′P read coverage relative to ORF start and stop codon. Cells were grown under different growth conditions. Exponentially growing cells at a lower cell density (OD_600_ ~ 0.3) are shown in purple and early stationary phase in green. (D) Relative 3-nt periodicity for the last 12 codons clustered using k-means. Each point corresponds to the ratio at each codon (ratio between peaks and valleys), not individual 5′P coverage as in (B). −17 nt indicates a ribosome paused at the stop codon and −50 nt corresponds to disome protection. The stop codon usage is represented in red and amino acids usage before stop codons (K, R, L, F) in blue.

An additional advantage of HT-5Pseq is that, by using a combination of random hexamers and oligo(dT) primers during reverse transcription, it enables control of the sequencing depth around the stop codon (Figure 3A). To further increase the coverage around the stop codon, we focused on those HT-5Pseq reads primed by oligo(dT) (Figure 3A). When analyzing the sequence coming from read 2 (Figure 1A), we can observe a clear bimodal distribution regarding the number of continuous As sequenced (Figure S3A). As expected, a fraction of reads originates from random hexamer priming (<5 As), whereas another fraction originates from oligo(dT) primed cDNA. Interestingly, in some cases we observed more than 10 As, suggesting that oligo(dT) priming could also occur downstream on the remaining poly(A) tail. This result also confirms the existence of a fraction of mRNA degradation intermediates containing sizable poly(A) tails. We used the information from read 2 to classify those reads primed by random hexamer (<5 As) or oligo(dT) (>9 As). As expected, 5′P degradome reads in the 3′ region of the genes are enriched in oligo(dT)-primed molecules (Figure 3A). It is important to note that altering the ratio of random hexamers and oligo(dT) primers will alter the relative coverage in 3′ regions of the mRNAs. However, in HT-5Pseq we always compare the changes in the 3-nt periodicity with its surrounding area, correcting it thus by its own sequencing coverage. Oligo(dT)-primed HT-5Pseq reads enabled the observation of a clear protection pattern associated with a single (−17 nt) and two ribosomes (−47/−50 nt) stalled at the stop codon. This could be observed both at metagene (Figure 3A) and single-gene level (Figure 3B). Using gene-specific information, we classified mRNAs according to their relative protection at the stop codon (−17) in respect of the upstream HT-5Pseq coverage as displaying low or high termination pausing (Figure 3B). When focusing on genes with sufficient coverage, we observed similar termination pauses on comparing random hexamer and oligo(dT) priming events (Figure S3B). Next, we investigated the relative expression of genes with a high observed termination pause, and found that highly expressed genes present an overall decrease in termination pause (Figure S3C). In agreement, mRNAs presenting low termination pauses are enriched for highly expressed gene ontology (GO) terms related to translation (e.g., GO:0002181, p_adjusted_ < 4.8 × 10^−9^) (Table S3). On the contrary, those associated with the transcription process had in general increased termination pausing (e.g., GO:0006355, p_adjusted_ < 6.2 × 10^−3^) (Table S3).

Having described degradation-associated ribosome stalls at the termination level under standard growth conditions (YPD [see STAR Methods], OD_600_ ∼ 0.6–0.8), we decided to investigate the regulation of this phenomenon. We have previously shown that during the early stationary phase (60 h), budding yeast displays a massive increase in ribosome stalls at termination level (Pelechano et al., 2015). Thus, we decided to compare the degradation-associated ribosome stalls in the early stationary phase and exponentially growing cells at a lower cell density (OD_600_ ∼ 0.3). In both cases we observed a clear stop pause at the stop codon (−17). The ribosome pausing of cells at early stationary phase was not only limited to −17 nt but also −47/−50 nt (corresponding to disomes) and −77/−80 nt (corresponding to trisomes) (Figure 3C), whereas exponentially growing cells at lower cell density (OD_600_ ∼ 0.3) presented a very subtle disome peak. Next, we investigated whether those pauses were differentially regulated at the single-gene level. As ribosome pausing was clearly observed at monosome, disome, and trisome level (from −80 to −17 nt), we clustered genes according to their relative pausing over the 22 last codons as measured by HT-5Pseq (Figure 3D). We clustered the observed gene-specific ribosome pauses in four groups. The first two clusters had more HT-5Pseq coverage in low-OD cells, suggesting a higher gene expression in that condition. Genes in cluster 1 displayed a clear translation termination pausing (−17), whereas genes in cluster 2 did not. Genes with high translation termination paused in cluster 1 were enriched in RNA metabolic process (GO:0016070, p_adjusted_ < 0.02), whereas genes with lower translation termination paused in cluster 2 were enriched in cytoplasmic translation (GO:0002181, p_adjusted_ < 7.1 ×10^−30^), similar to what we observed in exponentially growing cells at a higher OD (Table S4). On the contrary, genes in cluster 3 and 4 had more pronounced termination pausing (including disomes) in the early stationary phase. Genes in cluster 3 were enriched in aerobic respiration (GO:0009060, p_adjusted_ < 3.5 × 10^−9^), whereas genes in cluster 4 were associated with transcription (GO:0006355, p_adjusted_ < 7.5 × 10^−4^) (Table S4). By investigating mRNA features associated with different clusters, we found that those mRNAs in cluster 2 with lower termination pausing have in general a higher codon stability index, whereas clusters 3 and 4 with high termination pause in the early stationary phase show no differences in 3′ UTR length and mRNA half-life (Figures S3D–S3F). In summary, our results show that translation termination pausing associated with RNA decay is regulated according to cell growth. As ribosome stalling is tightly coupled with mRNA degradation, we decided to investigate to what degree mRNA degradation, termination pausing, and other mRNA features were related.

### Stop codon context affects termination pausing associated with mRNA decay

To investigate the relationship between ribosome pausing at the termination level and co-translational decay, we compared HT-5Pseq with alternative measurement of ribosome occupancy such as ribosome profiling (Guydosh and Green, 2014) or disome-seq (Meydan and Guydosh, 2020). We investigated whether features associated with termination pauses were shared among those methods that investigate different, though partially overlapping, subsets of ribosome-protected mRNAs.

**Figure 4:**
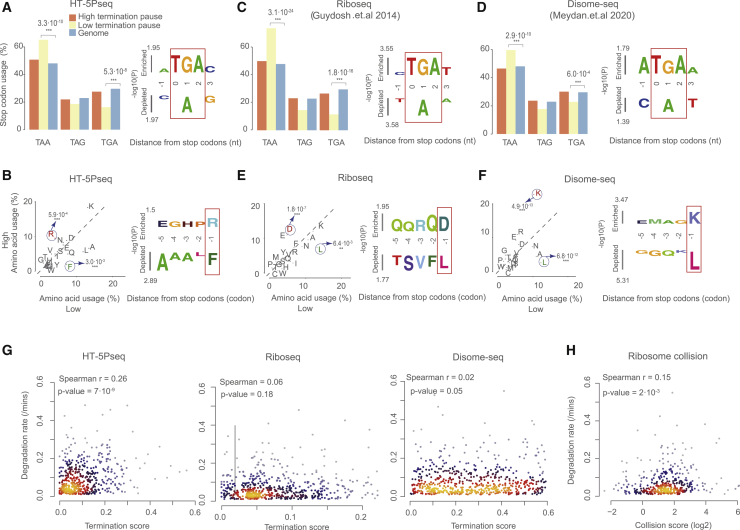
Stop codon context at termination pausing associated with mRNA decay (A) Frequency of stop codons usage in genes (left) and significance of enrichment for particular nucleotides at each position relative to stop codon (right) using kpLogo (Wu and Bartel, 2017) by comparing high and low termination pausing groups in HT-5Pseq. Significant deviation from genome average was estimated using a hypergeometric test; only significant p values are shown. (B) Frequency of last amino acid usage in genes with high termination pausing (y axis) versus low termination pausing (x axis) (left) and significance of enrichment for particular amino acids at each position relative to stop codon (right) as in (A). (C and D) As (A) but for (C) ribosome profiling data and (D) disome profiling data from Guydosh and Green (2014) and Meydan and Guydosh (2020). (E and F) As (B) but for (E) ribosome profiling data and (F) disome profiling data. (G) Spearman correlation between RNA degradation (calculated by total mRNA half-life from Presnyak et al., 2015) with termination pause in HT-5Pseq (left), ribosome profiling (middle), and disome profiling (right) data. (H) Spearman correlation between RNA degradation with collision scores. Collision scores were calculated from the disome profiling dataset by dividing disome peaks and monosome peaks of terminations stalling at stop codons.

We first defined a simple metric to quantify termination pausing across datasets and investigated pauses identified by HT-5Pseq. We quantified the termination pause as the relative protection by a ribosome paused at the stop codon (e.g., 17 nt upstream in the case of HT-5Pseq) and corrected this for gene-specific coverage upstream of that region (see STAR Methods for details). After ordering genes according to their level of termination pause, we classified genes with high or low termination pause as we defined previously (Figure 3B). In HT-5Pseq, genes with high termination pause present stop codon usage similar to that of the genome average (Figure 4A), whereas genes with lower termination pausing present an increased usage of TAA (65.1%, compared with the genome average of 47.5%) and decreased usage of TGA (16.3%, compared with the genome average of 29.6%). Observed termination pause was also influenced by the last amino acids preceding the stop codon (Figure 4B). Hydrophobic amino acids preceding the stop codons seem to associate with a decrease in termination pausing. Particularly, we found that phenylalanine (F) was enriched in genes with low termination pause, as well as other amino acids with hydrophobic chains (e.g., leucine [L] and alanine [A]), whereas the positively charged arginine (R) was enriched in genes with high termination pause (Figure S4A).

To investigate whether the same features were associated with general termination pausing or whether they were preferential for degradation-associated ribosome stalls, we repeated the same analysis with ribosome profiling and disome profiling data. We first compared the measured termination pause between HT-5Pseq, ribosome profiling, and disome-seq. In both ribosome profiling and disome-seq, the stop codon usage was similar to the one observed in HT-5Pseq (Figures 4C and 4D). This suggests that the degradation-associated ribosome stalls identified by HT-5Pseq share features with stalls identified by methods such as ribosome profiling and disome-seq. Next, we focused on the amino acid context upstream of the stop codon. In ribosome profiling, which can be expected to represent the global pool of ribosome-associated mRNAs, lysine was not enriched in genes with high termination pause. Instead, amino acids with negatively charged sides (i.e., aspartic acid [D]) were enriched in mRNAs with high termination pause (Figure 4E). However, arginine was enriched before the stop codon in mRNAs with low termination pause as in HT-5Pseq (Figure 4B). We then reanalyzed disome profiling datasets that would represent those ribosome stalls long enough to induce ribosome collisions, independent of their ability to induce mRNA decay (Meydan and Guydosh, 2020). In disome-seq, we found that lysine was clearly enriched in mRNAs with high termination pause (Figures 4F and S4A). Taking this information together, our results suggest that amino acid-specific preference associated with termination pauses might influence both the degree of termination pausing and their potential resolution and decay, and that degradation-associated ribosome stalls are more similar to those stalls able to induce ribosome collisions.

Finally, we investigated whether translation termination pauses as measured by HT-5Pseq were more likely to be associated with mRNA decay than stalls measured by different approaches. Specifically, we investigated the potential association between translation termination pausing and mRNA degradation (Presnyak et al., 2015). As expected, we found mild but significant correlation of termination stalling with mRNA degradation rate in HT-5Pseq (*ρ* = 0.26, p = 7.0 × 10^−9^) whereas the correlation was almost non-existent for general ribosome profiling (*ρ* = 0.06) as well as in disome-seq (*ρ* = 0.02, p = 0.05) (Figure 4G). This supported our initial hypothesis that translation termination pause measured by HT-5Pseq is likely enriched in those termination stalls that cannot be resolved, thus leading to increased mRNA decay. To further investigate this phenomenon by using an alternative dataset, we calculated “collision scores” of termination stalling by dividing disome peaks and monosome peaks from Meydan and Guydosh (2020). This measure reflects the relative enrichment of disome-associated stalls with respect to the intrinsic ribosome-associated stalls for each gene (Figures S4B and S4C). As expected, the collision score displayed an increased positive correlation of ribosome collisions with mRNA degradation (*ρ* = 0.15, p = 2.0 × 10^−3^). This suggests that at least part of the translation termination pauses caused by ribosome collision might be associated with decay. To further investigate this process, we analyzed the association of the measured termination pauses with other features associated with translation and decay (Carneiro et al., 2019). By analyzing HT-5Pseq data, we found that those mRNAs with lower termination pausing have in general higher codon stability index, codon optimality, and translation efficiency (Figures S4D–S4F). This effect was even more clear when focusing on the subset of transcripts by using TAA as stop codon. Our results show that by investigating ribosome termination pauses of different subsets of ribosomes, it is possible to obtain complementary information that can help us to better understand the functional consequences of ribosome stalls.

## Discussion

We have presented here an optimized protocol for the easy measurement of 5′-phosphorylated mRNA degradation intermediates, HT-5Pseq. HT-5Pseq has multiple advantages in respect of our original 5Pseq protocol (Pelechano et al., 2016), such as direct addition of sequencing adaptors during the reverse transcription step and avoiding the use of costly depletion of rRNA based on biotinylated oligonucleotides. This improved protocol decreases hands-on time and cost, and enables the easy handling of tens to hundreds of samples and potentially even more if combined with early multiplexing and sample pooling. In addition, by eliminating unnecessary purification steps it lowers the required input material from the 10–100 μg recommended for original 5Pseq. Although in this article we demonstrate HT-5Pseq applicability by using 6 μg of total RNA, we have successfully performed HT-5Pseq libraries with as little as 500 ng of total RNA. We have shown that HT-5Pseq is a simple and reproducible approach to detect codon-specific ribosome stalling in both *S*. *cerevisiae* and *S*. *pombe* and that it is especially well suited to investigate those ribosome stalls that frequently lead to RNA decay.

Next, we have also investigated the regulation and relevance for mRNA decay of *in vivo* co-translational mRNA degradation footprints associated with ribosome stalling at the termination level. It is known that interaction between the ribosome and mRNA sequences nearby the stop codon could affect termination pausing. In some cases, this can be attributed to the interaction of ribosomes with positive lysine amino acid before the stop codon, which hinders the binding of the release factors. In addition, termination stalling can also be enhanced by interfering with the translation termination process, for example in strains depleted for eIF5A or Rli/ABCE (Young et al., 2015; Pelechano and Alepuz, 2017; Schuller et al., 2017). Here, we showed the mRNAs presenting lower termination pauses use more frequently TAA as stop codons. To investigate whether amino acid usage before the stop codon was associated with ribosome stalling and whether this was different between different pools of ribosome-associated mRNAs, we calculated the frequency of amino acid usage in HT-5Pseq, ribosome profiling, and disome-seq. We found that positively charged amino acids (i.e., lysine and arginine) are enriched in HT-5Pseq and disome-seq with high termination pausing. On the contrary, negatively charged amino acids such as aspartic acid are enriched in ribosome profiling with high termination pausing. We propose that these differences can be explained by the fact that each method measures a differential subset of ribosomes that differ in relative ribosome stalling, likelihood of ribosome collision, and association with co-translational mRNA decay.

Finally, to directly investigate the relationship between co-translational mRNA decay as measured by HT-5Pseq and mRNA stability, we compared ribosome pausing at stop codons with mRNA degradation rates. As HT5-Pseq focuses on those mRNAs undergoing degradation, we hypothesized that it would be especially useful to identify those ribosome stalls associated with mRNA decay. As expected, we found a significantly positive correlation between ribosome stalls measured by HT-5Pseq and mRNA degradation rates. However, this was not the case for ribosome profiling or disome-seq data. This suggests that those stalls identified by HT-5Pseq are more likely to be associated with mRNA decay than those observed by different approaches. However, the functional consequence of those stalls could not be observed when investigating disome or monosome pauses independently. As HT-5Pseq focuses exclusively on the subset of ribosomes undergoing co-translational degradation, we think that it offers complementary information to general approaches such as ribosome profiling. By using the *in vivo* toeprinting activity of Xrn1p, HT-5Pseq can easily capture disomes or trisomes *in vivo*. Although disomes can also be captured by using specialized ribosome profiling approaches, such as disome-seq (Zhao et al., 2021; Han et al., 2020), depending on the stringency of the used *in vitro* RNase I treatment, ribosome footprints can be altered (Mohammad et al., 2019). This can lead to the loss of some disome complexes (e.g., those separated by an extra codon) (Zhao et al., 2021). Additionally, as it has been reported that not all disome events lead to mRNA decay (Meydan and Guydosh, 2020), disome profiling offers different biological information. In summary, we think that HT-5Pseq will be a valuable tool to facilitate the investigation of mRNA life from translation to decay, and will be especially useful to functionally characterize those ribosome stalls leading to mRNA decay. We expect that, by combining multiple genomic and structural approaches, in the future we will be able to understand better this fundamental process in biology.

### Limitations of the study

Although HT-5Pseq enables the identification of degradation-associated ribosome stalls, this approach has some limitations. First, HT-5Pseq focuses on the subpopulation of 5′ end of mRNA undergoing co-translational decay, and not on the bulk of translating ribosomes. Thus, we do not recommend its use to estimate translation rates. In those conditions, general methods such as ribosome profiling, or even better proteomic based approaches, would be preferable. Second, as in eukaryotes co-translation decay is shaped by the 5′-3′ exonuclease Xrn1p, extreme caution should be taken when interpreting profiles of cells with altered RNA degradation. However, this limitation is an advantage when trying to understand the impact of translation elongation or ribosome recycling pathways on mRNA. HT-5PSeq is also well suited to study the crosstalk between mRNA surveillance pathways such as non-sense mediated decay (NMD) or no-go decay (NGD) and ribosome dynamics.

## STAR★Methods

### Key resources table

**Table:** 

REAGENT or RESOURCE	SOURCE	IDENTIFIER
**Chemicals**, **peptides**, **and recombinant proteins**		

Ethanol absolute ≥99.8%	VWR	20821.330
Glass beads, acid-washed	Sigma-Aldrich	G8772
Acid-Phenol:Chloroform, pH 4.5 (with IAA, 125:24:1)	Thermo Fisher Scientific	AM9722
dNTP set, 100mM solution	Thermo Fisher Scientific	R0181
Phenol Solution. Saturated with 0.1M citrate buffer, pH 4.3 ± 0.2	Sigma-Aldrich	P4682
Chloroform:isoamyl alcohol 24:1	Sigma-Aldrich	C0549
Sodium Acetate buffer solution, pH 5.3	Sigma-Aldrich	S7899
Glycoblue coprecipitant (15mg/mL)	Thermo Fisher Scientific	AM9515
Nuclease-free water, not DEPC treated	Thermo Fisher Scientific	AM9937
Ribolock RNase inhibitor 40 000U/mL	Thermo Fisher Scientific	EO0382
Turbo Dnase kit	Thermo Fisher Scientific	AM1907
T4 RNA ligase 1	NEB	M0204L
SuperScript™ II Reverse Transcriptase	Thermo Fisher Scientific	18064071
Phusion®High-Fidelity PCR Master Mix	NEB	M0531S
AMPure XP	Beckman Coulter	A63881
RNAClean XP	Beckman Coulter	A63987
Duplex-specific nuclease	Evrogen	EA002

**Critical commercial assays**		

High-sensitivity DNA kit	Agilent	5067-4626
Qubit™ dsDNA HS Assay Kit	Thermo Fisher Scientific	Q32854
Qubit™ RNA HS assay kit	Thermo Fisher Scientific	Q32852

**Deposited data**		

The raw and processed sequencing data	This paper	GEO: GSE152375

**Experimental models**: **organisms/strains**		

*Saccharomyces cerevisiae* strains: BY4741:(MATa *his3Δ1 leu2Δ0 met15Δ0 ura3Δ0*)	NA	NA

**Oligonucleotides**		

See Table S1	This paper	N/A

**Software and algorithms**		

*Fivepseq* package	Github	Nersisyan et al., 2021
bcl2fastq v2.20.0	Illumina	https://emea.support.illumina.com/sequencing/sequencing_software/bcl2fastq-conversion-software.html
Cutadapt	GitHub	https://github.com/marcelm/cutadapt/
UMI-tools	GitHub	Smith et al., 2017
STAR 2.7.0	GitHub	Dobin et al., 2013
DESeq2	Bioconductor	Love et al., 2014
Subread package	GitHub	Liao et al., 2014
IGV	https://igv.org	Thorvaldsdóttir, 2013
RStudio Version 3.5.0	RStudio, Inc., Boston, MA	N/A

### Resource availability

#### Lead contact

Further information and requests for resources and reagents should be directed to and will be fulfilled by the Lead Contact, Vicent Pelechano (vicente.pelechano.garcia@ki.se).

#### Materials availability

This study did not generate new unique materials nor reagents.

#### Data and code availability

The raw and processed sequencing data are deposited at GEO with accession number GSE152375.

### Experimental model and subject details

*Saccharomyces cerevisiae strain* BY4741 (MAT a *his3Δ1 leu2Δ0 met15Δ0 ura3Δ0*) was grown to mid exponential phase (OD_600_∼0.8) at 30°C using YPD (1% yeast extract, 2% peptone, 2% glucose). *Schizosaccharomyces pombe* (h-) was grown at 30°C to mid-log phase (OD600 ∼0.8) using YES media (0.5% yeast extract, 3% glucose, supplemented with 225 mg/l of adenine, histidine, leucine, uracil, and lysine). For cycloheximide (CHX) treatment, CHX was added to final 0.1 mg/mL to the medium and incubated for 5 min at 30°C for *S*. *cerevisiae* and 10 min for *S*. *pombe*. For early exponential phase, strains were grown at 30°C from an initial OD_600_∼0.05 to a final OD_600_ of 0.3. To reach early stationary phase, *S*. *cerevisiae* strains were grown during 60 h in YPD. All yeast samples were collected by centrifugation and pellets were frozen in liquid nitrogen. We used 2 mL culture and harvested the cells using a benchtop microcentrifuge (30 sec at 8.000xg). It is important to minimize time for harvesting cells as it is known that ribosome positions can be altered during sample handling. Total RNA was isolated by the standard phenol:chloroform method, and DNA was removed by DNase I treatment. RNA integrity was checked by agarose gel.

### Method details

#### HT-5Pseq library preparation and sequencing

For HT-5Pseq libraries construction, we used 6 μg of DNA-free total RNA. Samples were directly subjected to RNA ligation. The treated RNA samples were incubated with 100 μM RNA rP5_RND oligo (final 10 μM, Table S1B) 2 h at 25°C with 10 Units of T4 RNA ligase 1 (NEB). Please note that we used an RNA oligo, and not the DNA/RNA oligonucleotide previously used (Pelechano et al., 2016). Ligated RNA was purified with RNA Clean XP (Beckman Coulter), according to the manufacturer’s instructions. RNA was reverse transcribed with Superscript II (Life Technologies) and primed with Illumina PE2 compatible oligos containing random hexamers (20 μM, Table S1B) and oligo-dT (0.05 μM, Table S1B). This enables the capture of the most 3′ region that is loss if only random hexamers are used (Xu et al., 2009). Reverse transcription reaction was incubated for 10 min at 25°C, 50 min at 42°C and heat inactivated for 15 min at 70°C. To deplete RNA in RNA/cDNA hybrid after reverse transcription, we used sodium hydroxide (40 mM) for incubation 20 min at 65°C and then neutralized with Tris-HCl, pH =7.0 (40 mM). For DSN (Duplex-specific nuclease) based rRNA depletion, we used a mixture of probes (Table S1A) targeting the 18S rDNA, 25S rDNA and 5.8S rDNA. The probes were designed to occupy the whole ribosomal RNA regions with consecutive 25–30 nt long unmodified DNA oligos. The hybridization of probes (2 μM each) with cDNAs were incubated at 68°C for 2 min before adding pre-warmed DSN buffer mix with 1 Units of DSN enzyme (Evrogen). The reaction then performed at 68°C for 20 min. To inactive DSN enzyme, we added 2X DSN stop solution and incubate 10 min at 68°C. The final PCR amplification was performed using 2X Phusion High-Fidelity PCR Master Mix with HF Buffer (NEB) and final 0.1 μM of PE1.0 and corresponding multiplex PE2.0_MTX (Table S1B). The program followed this: 30s 98°C; 15 cycles (20s 98°C; 30s 65°C; 30s 72°C); 7min 72°C. Libraries were size selected using 0.7x–0.9x (v/v) AMpure XP beads (Beckman Coulter) to final length of 200–500 bp and sequenced by NextSeq 500 using 60 sequencing cycles for Read 1 and 15 cycles for Read 2.

#### Read preprocessing and analysis

3′-sequencing adaptor trimming was applied to 5′ends of reads using cutadapt V1.16 (http://gensoft.pasteur.fr/docs/cutadapt/1.6/index.html). The 8-nt random barcodes on the 5′ ends of reads were extracted and added to the header of fastq file as the UMI using UMI-tools (Smith et al., 2017). Reads were mapped to the reference genome (SGD R64-1-1 for *S*. *cerevisiae* genome, ASM294v2.20 for *S*. *pombe* genome) separately by star/2.7.0 (Dobin et al., 2013) with the parameter --alignEndsType Extend5pOfRead1 to exclude soft-clipped bases on the 5′ end. To calculate the fraction of rRNA, tRNA, snRNA snoRNA and mRNA in library compositions, the stepwise alignment was performed by corresponding index generated by star/2.7.0. Duplicated 5′ ends of read introduced by PCR during library preparation were removed based on random barcodes sequences using UMI-tools (Smith et al., 2017). To compare the differences of 5′P read coverage in DSN based rRNA depletion at gene level, reads per gene were counted using Subread package (featureCounts) (Liao et al., 2014). mRNA, tRNA, rRNA and snRNA and snoRNA transcripts were counted separately and combined for further analysis. Differential gene expression analysis were performed using the DESeq2 (Love et al., 2014) packages from R and Bioconductor (http://www.bioconductor.org/) (Love et al., 2014). The threshold for differentially expressed genes were defined as p value < 0.005 and log2(fold-change) > 1. Analysis of 5′ ends positions was performed using *Fivepseq* package (Nersisyan et al., 2020) (http://pelechanolab.com/software/fivepseq), including relative to start, stop codon and codon specific pausing. Specifically, the unique 5′mRNA reads in biological samples were summed up and normalized to reads per million (rpm). Then the relative position of 5′mRNA reads to all codons of all ORF were summed at each position. Metagene plots were showed as the sum value versus the relative distance from respective codon. All clustering analyses was performed by k-means using Complexheatmap packages from R and Bioconductor (Gu et al., 2016). Dataset for *S*. *cerevisiae* tRNA adaptation index, mRNA codon stability index, translation efficiency were obtained from Carneiro et al. (Carneiro et al., 2019), gene expression level and mRNA half-life were obtained from Xu et al. (Xu et al., 2009) and Presnyak et al. (Presnyak et al., 2015), respectively.

### Quantification and statistical analysis

Gene Ontology enrichment analysis in Tables S3 and S4 were performed with ClusterProfiler using Fisher’s exact test (Yu et al., 2012). Significance for enrichment for particular nucleotides and amino acids at each position were performed using kpLogo (Wu and Bartel, 2017). Significant deviation of stop codon usage in high or low termination pausing from genome average was estimated using hypergeometric test (Figure 4). Ribosome profiling and Disome-seq data were obtained from Guydosh and Green (Guydosh and Green, 2014) and Meydan and Guydosh (Meydan and Guydosh, 2020), respectively. Termination pause scores were calculated in each dataset by dividing the peaks from termination versus the upstream 27-nt regions (before the following ribosome queuing). Collision scores were calculated from Meydan and Guydosh (Meydan and Guydosh, 2020) by dividing termination pause scores in disome versus monosome profiling.

## Supplemental information

Document S1. Figures S1–S4

Table S1. (A) rRNA depletion oligos, related to STAR Methods; (B) Oligos used in HT-5Pseq, related to Figure 1

Table S2. HT-5Pseq library cost estimates, related to Figure 1

Table S3. Genes and gene ontologies associated with high and low termination pauses, related to Figure 3

Table S4. Genes and gene ontologies associated with high and low termination pauses during growth condition, related to Figure 3

Document S2. Article plus supplemental information
