# Supplementary material for: High-throughput 5′P sequencing enables the study of degradation-associated ribosome stalls
Source: Cell Rep Methods. 2021 Apr 2;1(1):100001. doi: 10.1016/j.crmeth.2021.100001 (PMC9017187; doi:10.1016/j.crmeth.2021.100001)
Supplement: Document S1. Figures S1–S4 [file mmc1.pdf]

Cell Reports Methods, Volume *1*

Supplemental information

High-throughput 5′P sequencing enables the study of degradation-associated ribosome stalls

Yujie Zhang and Vicent Pelechano

Supplementary material

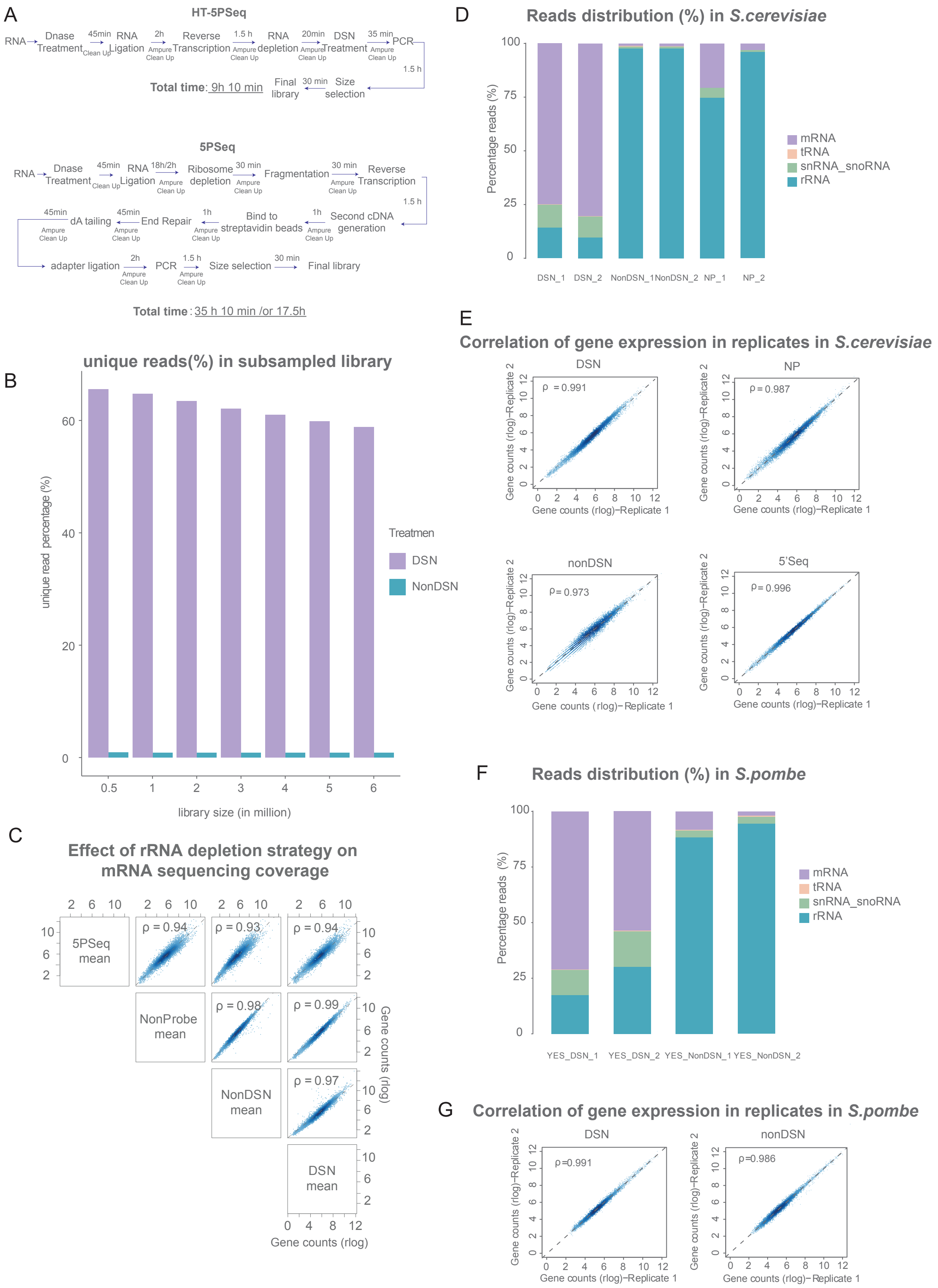

Figure S1. Performance and reproducibility of HT-5Pseq, Related to Figure 1. (A) Flowchart of HT-5Pseq and 5PSeq with time estimate for each step in details. (B) mRNA sequencing library complexity analysis subsampling libraries for HT-5Pseq and control omitting rRNA depletion (NonDSN). Error bars indicate standard errors of the two replicates libraries. (C) Effect of rRNA depletion strategy on mRNA sequencing coverage. Spearman correlation between 5PSeq (Pelechano, Wei and Steinmetz, 2015) and HT-5Pseq is shown. NonDSN refers to control libraries omitting DSN rRNA depletion. NonProbe refers to libraries treated with DSN but omitting the depletion probes. 5´P read gene coverage (in coding region) shown in rlog. (D) Improvement of mRNA mapability in *S.cerevisiae* HT-5Pseq after rRNA depletion. NonDSN refers to control libraries omitting DSN rRNA depletion. NonProbe refers to libraries treated with DSN but omitting the depletion oligos. 2 biological replicates are shown. (E) Spearman correlation for mRNA sequencing coverage comparing biological replicates for HT-5Pseq, NonProbe, NonDSN and traditional 5PSeq. 5PSeq (Pelechano, Wei and Steinmetz, 2015) and HT-5Pseq. NonDSN refers to control libraries omitting DSN rRNA depletion. NonProbe refers to libraries treated with DSN but omitting the depletion oligos. 5´P read gene coverage shown in rlog. (F-G) Differential gene-specific 5´P read coverage. (F and G) as D and E but for *S. pombe.*

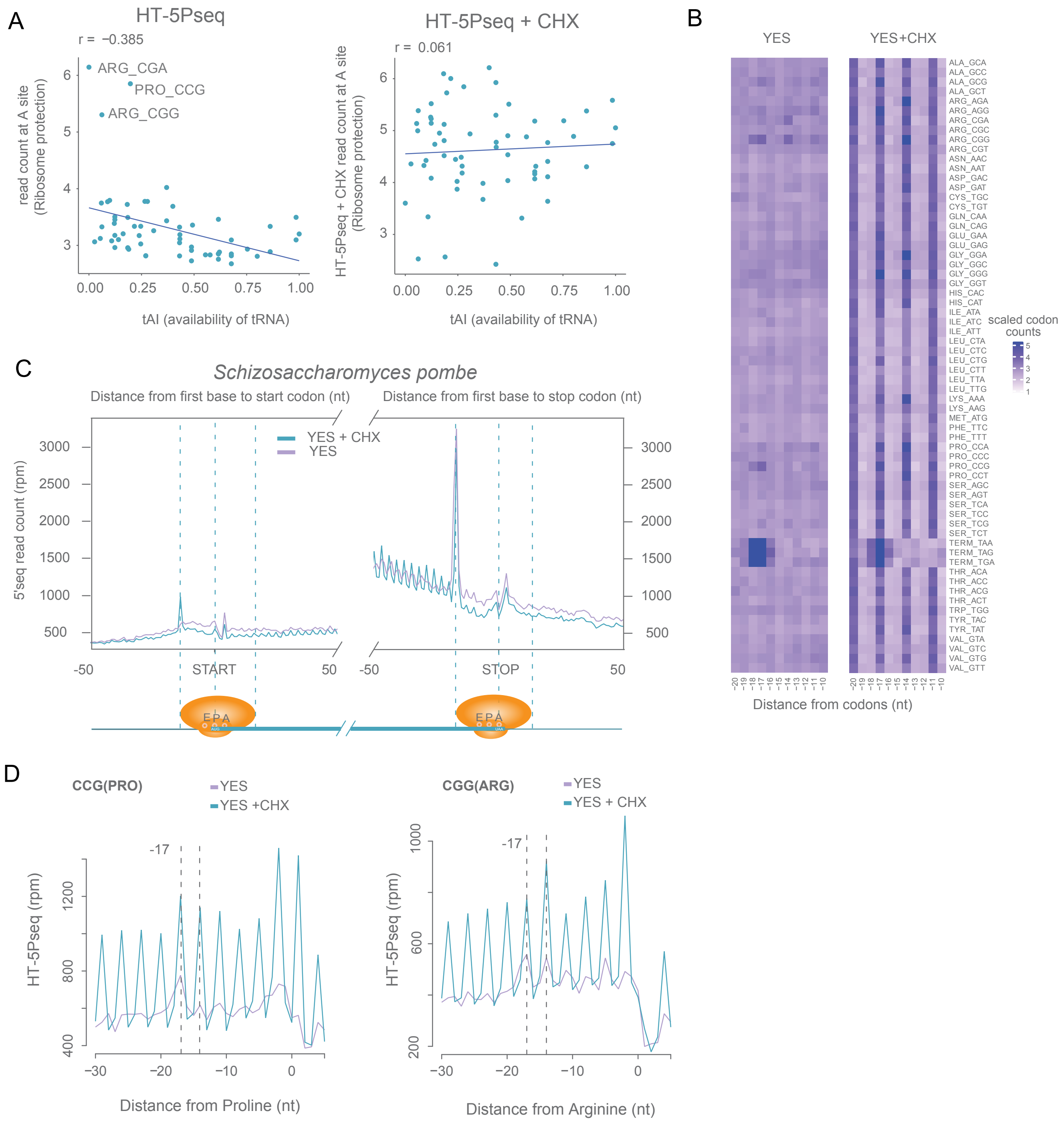

Figure S2. HT-5Pseq reveals ribosome dynamics in *S. pombe,* Related to Figure 2. (A) Correlation of 5´P read abundance at A site with tAI (tRNA adaptation index) with or without CHX for *S.cerevisiae.* (B) Heatmap of average codon coverage for cells grown in rich media (YES) and CHX treatment (YES + CHX) in *S.pombe*. 5´P reads relative to each codon were summed up and normalized to the reads corresponding to the surrounding (-30 to 5 nt). -17, -14 and -11 represent A site, P site and E site on ribosome. (C). Metagene analysis showing the abundance of 5´P reads relative to ORF start codons and stop codons for wild type in rich media (YES, in purple) and CHX treatment for 10 mins (YES + CHX, in blue) in *S.pombe*. (D) 5´P reads coverage for proline codons (CCG) and rare arginine (CGG). Dotted lines at -17 and -14 corresponding to the expected 5´ end of protected ribosome located at the A site or P site, respectively in *S. pombe*.

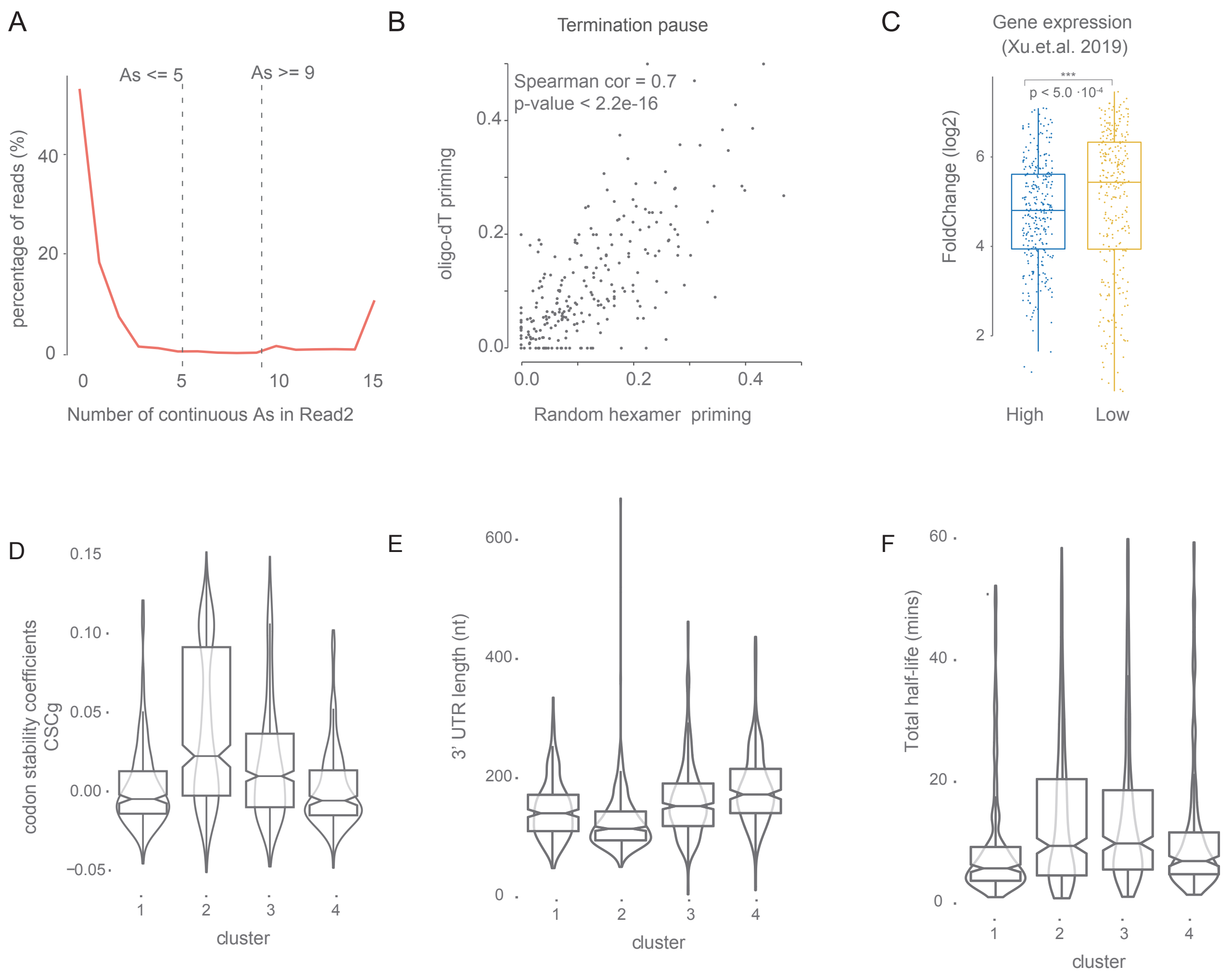

Figure S3. Influence of stop codon environment on translation termination pauses, Related to Figure 3. (A) The distribution of 5´P mRNA degradation intermediates with different number of continuous As in Read1. (B) Comparison of termination pauses between random hexamer and oligo-dT priming events in HT-5Pseq. (C) Boxplot for gene expression level (array intensity). Gene expression data was obtained from Xu.*et.al* (Xu *et al.*, 2009). High termination pause genes are shown in blue and low termination pause in yellow. P-values were calculated by the Wilcoxon Rank Test (two-tailed test). (D-F) Cluster specific codon stability coefficients (CSCg), 3‘UTR length and mRNA half life from Carneiro et al. (Carneiro *et al.*, 2019) , Pelechano et al. (Pelechano, Wei and Steinmetz, 2013) and Presnyak et al.(Presnyak *et al.*, 2015)

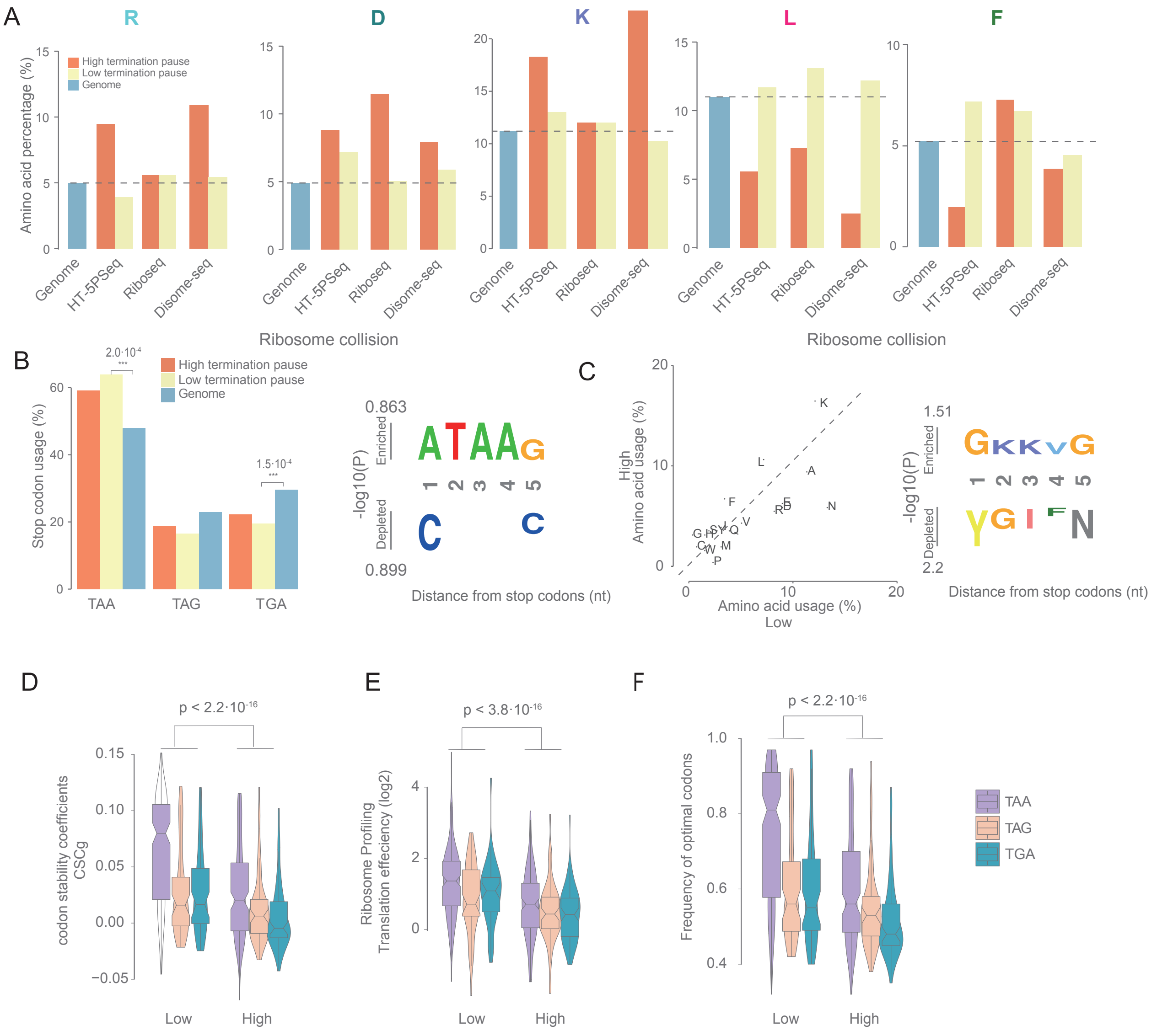

Figure S4. Characteristics of genes with differential ribosome termination pausing, Related to Figure 4. (A) Frequency of last amino acid usage (R, D, K, L, F) by comparing genes with high (in orange) and low termination (in yellow) pausing groups in HT-5Pseq, ribosome profiling and disome profiling data. Frequency of last amino acid usage across the genome is shown in blue. (B) Frequency of stop codons usage in genes (left) and significance for enrichment for particular nucleotides at each position relative to stop codon (right) using kpLogo (Wu and Bartel, 2017) by comparing high and low termination pausing groups in ribosome collision. Significant deviation from genome average was estimated using hypergeometric test, only significant p-values are shown. (C) Frequency of last amino acid usage in genes with high (y-axis) versus low termination pausing (x-axis) (left) and significance for enrichment for particular amino acid at each position relative to stop codon (right) as in B. Please note that error associated to collisions scores measure is higher as it combines disome-seq and ribosome profiling information. (D-F) Comparison of high and low termination groups in: (D) codon stability coefficients (CSCg), (E) frequency of optimal codons and (F) ribosome profiling translation efficiency from Carneiro et al. (Carneiro *et al.*, 2019). High and low termination pauses measured as in described in Figure 4 for HT-5PSeq, ribosome profiling (Guydosh and Green, 2014), disome profiling and ribosome collision (Meydan and Nicholas R. Guydosh, 2020). P-values were calculated by the Wilcoxon Rank Test (two-tailed test).
